# Knockdown of ZFAS1 improved the cardiac function of myocardial infarction rats via regulating Wnt/β-catenin signaling pathway

**DOI:** 10.18632/aging.202961

**Published:** 2021-05-05

**Authors:** Bing Zou, Tieqiu Huang, Dan Wu, Xiaomin Hu, Linghui Xiao, Chenxi Wang, Hongzhou Zhang, Jian Xiang, Chenkai Hu, Qinghua Wu, Tao Wu

**Affiliations:** 1 Department of Cardiovascular Medicine, The Second Affiliated Hospital of Nanchang University, Nanchang, Jiangxi 330006, China; 2 Jiangxi Health Vocational College, Nanchang, Jiangxi 330052, China

**Keywords:** long non-coding RNA, SERCA2a, cardiomyocytes, coronary artery disease, vWF

## Abstract

Myocardial infarction (MI) is a big health threat in the world, and it is characterized by high morbidity and mortality. However, current treatments are not effective enough, and novel therapeutic strategies need to be explored. ZFAS1 has been proved to be involved in the regulation of MI, but the specific mechanism remains unclear. MI rats were constructed through left anterior descending artery ligation, and hypoxia cell model was also established. The proliferation, invasion, and migration of cells were detected via CCK8, traswell, and wound healing methods. Immunohistochemistry staining, western blotting, and qRT-PCR were used to detect the levels of molecules. Knockdown of ZFAS1 significantly increased the proliferation, migration, and invasion of cardiac fibroblasts. Knockdown of ZFAS1 remarkably improved cardiac function via decreasing infarction ratio and increasing vWF expression, left ventricular ejection fraction, and left ventricular fractional shortening compared with group MI. Knockdown of ZFAS1 also suppressed Wnt/β-catenin pathway *in vivo*. The inhibition of Wnt/β-catenin remarkably reversed the influence of shZFAS1 on cardiac function and cardiac fibroblasts viability. Therefore, Knockdown of ZFAS1 could improve the cardiac function of myocardial infarction rats via regulating Wnt/β-catenin signaling pathway. The present study might provide new thoughts for the prevention and treatment of MI damage.

## INTRODUCTION

Myocardial infarction (MI) is a kind of myocardial necrosis caused by acute ischemia and hypoxia of coronary artery. If the coronary artery is blocked for more than 40 minutes, the myocardium will be damaged irreversibly [1, 2]. Some risk factors including smoking, drinking, high-fat and high calorie diet have been proved to be linked with MI [3]. At present, the treatments of MI include thrombolysis, intracoronary stent implantation, coronary artery bypass grafting and drug therapy [4]. The above treatments can improve symptoms, but the curative effect of some patients is poor. Although timely opening of occluded coronary artery by percutaneous coronary intervention (PCI) can significantly reduce the mortality of MI. However, a successful implementation of PCI is not effective for some patients, and the chest pain and other symptoms cannot be completely relieved [5]. Therefore, novel therapeutic strategy needs to be explored for the prevention and treatment of MI.

Long non-coding RNA (LncRNA) is a class of RNA molecules with transcripts longer than 200 bp and without the ability to encode mature proteins. A large number of studies have shown that LncRNA is involved in the occurrence and progression of tumor, diabetes, cardiovascular disease and other diseases [6, 7]. Zinc finger antisense 1 (LncRNA-ZFAS1, ZFAS1), the antisense strand of zinc lipoprotein coding gene Znfx1, is a long non-coding RNA [8, 9]. Previous study indicated that ZFAS1 could regulate cardiovascular system, and it is a potential diagnostic marker of acute myocardial infarction [10]. Meanwhile, ZFAS1is an endogenous inhibitor of sarcoplasmic reticulum Ca^2+^ ATPase 2A (SERCA2a), which can induce intracellular calcium overload in myocardial cells and further lead to systolic and diastolic dysfunction after myocardial infarction [11, 12]. In addition, high expression of ZFAS1 in myocardial cells after MI can activate the mitochondrial apoptotic pathway and induce myocardial cell apoptosis [12]. Silencing of ZFAS1 could protect against hypoxia/reoxygenation-induced injury [13] and ischemia/reperfusion-induced cardiomyocytes apoptosis via the miR-590-3p/NF-κB signaling pathway [14]. Therefore, ZFAS1 is a potential function target for the prevention and treatment of MI. However, the functional role of ZFAS1 in MI requires further clarification.

Wnt/β-catenin signaling pathway is involved in embryo and organ development. Wnt family genes mainly encode secretory signaling proteins, which are related to tumorigenesis and adipogenesis. In addition, it is related with the regulation of cell differentiation during embryonic development [15–17]. It was found that the expression of Wnt3a and Wnt5a was up-regulated in cardiomyocytes of mice with cardiac hypertrophy, which may be related to cardiomyocyte apoptosis. It was reported that Wnt/β-catenin pathway was involved in the healing process of acute myocardial infarction [18–20]. However, the specific mechanism how Wnt/β-catenin pathway to affect the healing process needs to be further elucidated. Meanwhile, if ZFAS1 could regulate MI through targeting Wnt/β-catenin has not been reported.

In this study, MI rats and hypoxia cell models were established. Knockdown of ZFAS1 was constructed and the influence of ZFAS1 on the cell viability and cardiac function were investigated. Meanwhile, the effect of ZFAS1 on the Wnt/β-catenin pathway and if ZFAS1 could regulate MI through Wnt/β-catenin were also studied. This study provides a new insight for the prevention and treatment of MI damage.

## RESULTS

### Knockdown of ZFAS1 significantly promoted the cell viability

**Figure 1:**
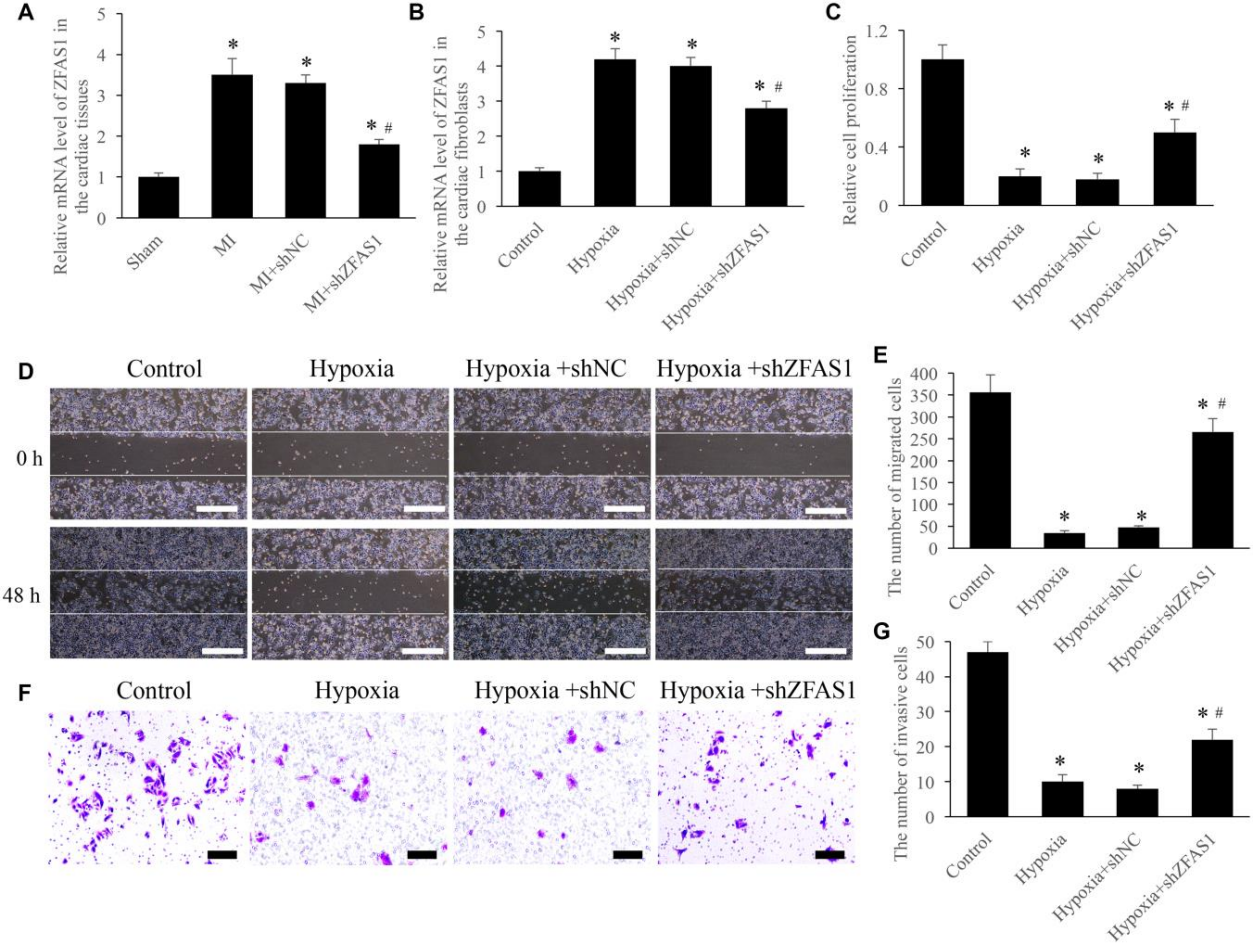
**Knockdown of ZFAS1 significantly promoted the cell viability.** (**A**) The expression of ZFAS1 was measured in the MI model. (**B**) The expression of ZFAS1 was measured in the hypoxia cell model. (**C**) Knockdown of ZFAS1 markedly promoted the ability of cell proliferation. (**D**) Cell migration was measured using wound healing method (Scale bar = 500 *μ*m). (**E**) Knockdown of ZFAS1 markedly promoted cell migration compared with group hypoxia. (**F**) Cell invasion was measured using Transwell method (Scale bar = 200 *μ*m). (**G**) Knockdown of ZFAS1 markedly promoted *t* cell invasion compared with group hypoxia. ^*^P < 0.05 compared with the group control or sham. ^#^*P* < 0.05 compared with the group hypoxia.

In order to investigate the role of ZFAS1 on MI, both *in vivo* and *in vitro* models were established. Significant increase of ZFAS1 in both MI and hypoxia models was observed (Figure 1A and 1B). However, knockdown of ZFAS1 remarkably decreased the level of ZFAS1 (Figure 1A and 1B). Meanwhile, the proliferation, migration, and invasion of CFs were significantly suppressed after hypoxia treatment (Figure 1C–1G). However, simultaneous treatment with shZFAS1 markedly reversed the influence of hypoxia treatment, promoted the proliferation, migration, and invasion ability of CFs (Figure 1C–1G). Therefore, ZFAS1 might act a key role during the process of MI.

### Improvement of cardiac function by silencing ZFAS1 in the MI rats

**Figure 2:**
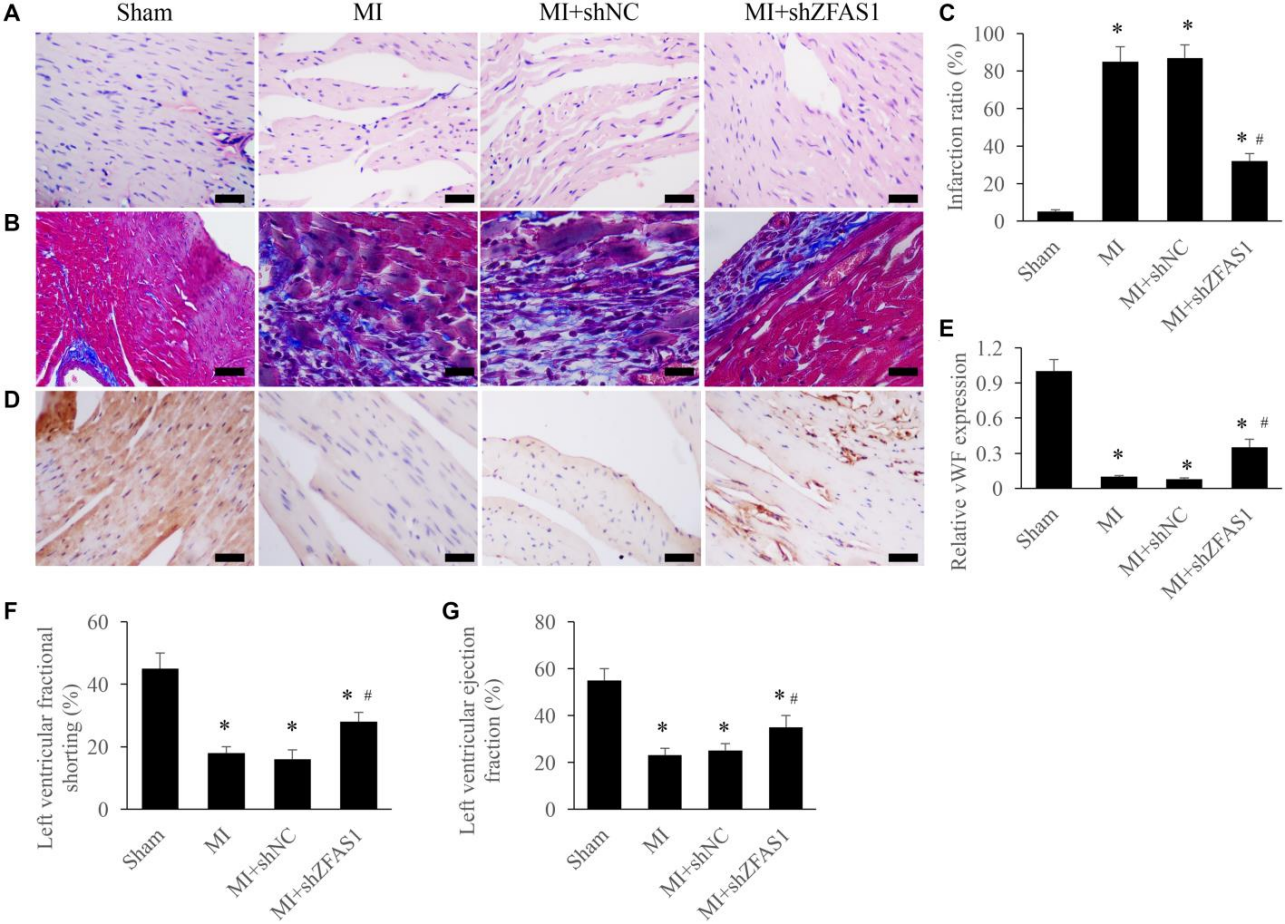
**Improvement of cardiac function by silencing ZFAS1 in the MI rats.** (**A**) Influence of shZFAS1 on the histological changes of MI rats myocardial tissues (Scale bar = 500 *μ*m). (**B**) Influence of shZFAS1 on the collagen deposition of MI rats myocardial tissues (Scale bar = 500 *μ*m). (**C**) Influence of shZFAS1 on the infarction ratio of MI rats myocardial tissues. (**D**) The expression of vWF in the MI rats myocardial tissues was measured using IHC staining (Scale bar = 500 *μ*m). (**E**) Influence of shZFAS1 on the vWF expression in the MI rats myocardial tissues. (**F**) Influence of shZFAS1 on the left ventricular fractional shortening of MI rats. (**G**) Influence of shZFAS1 on the left ventricular ejection fraction of MI rats. ^*^P < 0.05 compared with the group sham. ^#^*P* < 0.05 compared with the group MI.

Morphological changes of heart tissues were investigated using Masson and HE staining. After treatment with MI and MI+shNC, disorder arrangement of cardiomyocytes and enlargement of tissues gap were observed (Figure 2A). Meanwhile, remarkable increase of myocardial fibrosis measured by Masson staining was found in the group MI and MI+shZFAS1 (Figure 2B). Infarction ratio was analyzed by measuring the intensity of collagen deposition. MI treatment significantly increased infarction ratio compared to group sham, but knockdown of shZFAS1 markedly reversed the effect of MI, and decreased the level of infarction ratio (Figure 2C). In addition, the level of vWF in the tissues was detected via IHC to investigate the changes of angiogenesis. Remarkable lower levels of vWF in the group MI and MI+shNC were observed, but shZFAS1 significantly promoted the expression of vWF compared to these two groups (Figure 2D and 2E). In addition, in group MI and MI+shNC, both left ventricular ejection fraction (LVEF) and left ventricular fractional shortening (LVFS) were remarkably inhibited compared with group sham (Figure 2F and 2G). Similarly, LVEF and LVFS were markedly increased by shZFAS1. These findings indicate that knockdown of ZFAS1 could improve MI.

### Promotion of Wnt/β-catenin by shZFAS1 in the MI rats

**Figure 3:**
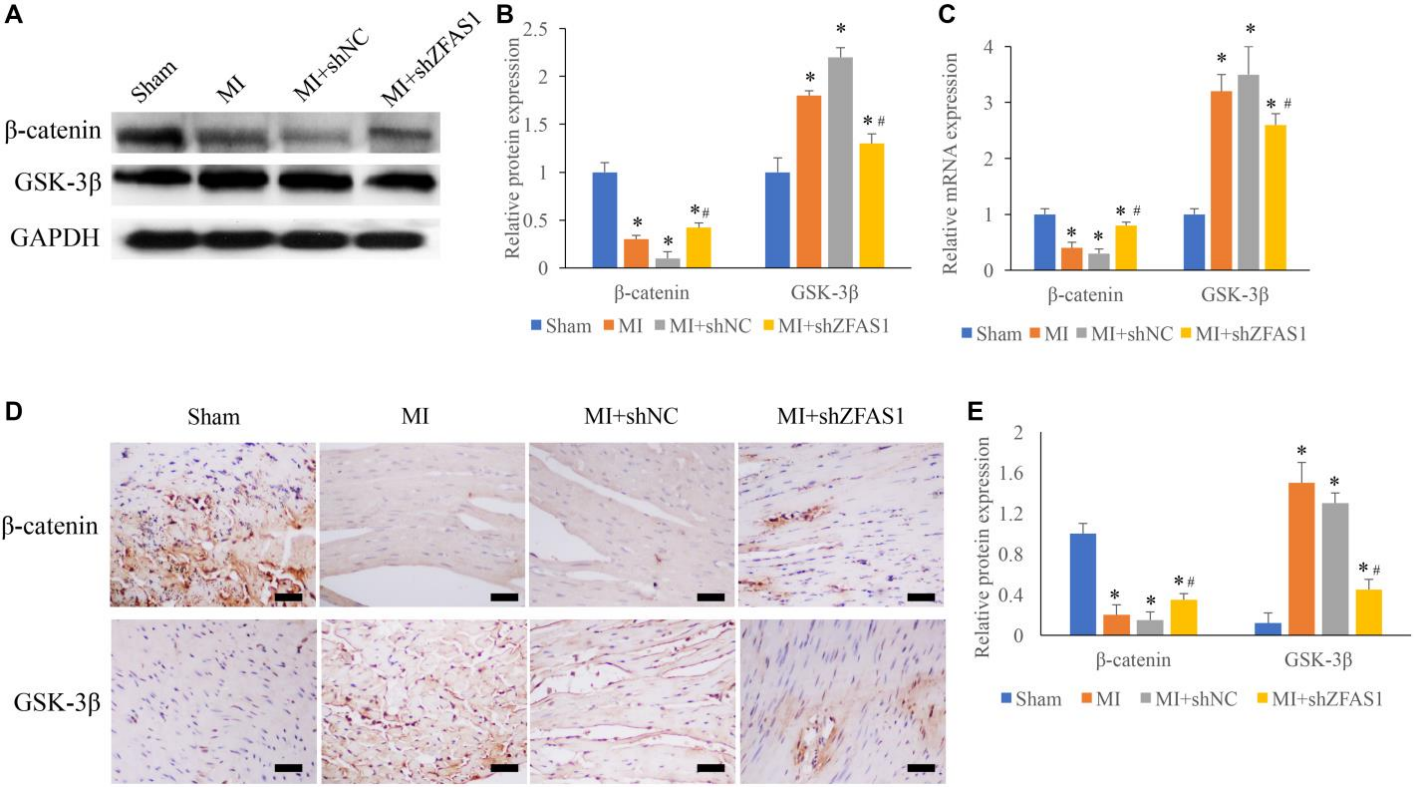
**Promotion of Wnt/β-catenin pathway by shZFAS1 in the MI rats.** (**A**) The protein expression of β-catenin and GSK-3β in the myocardial tissues was measured. (**B**) Influence of shZFAS1 on the protein expression of β-catenin and GSK-3β in the myocardial tissues. (**C**) Influence of shZFAS1 on the mRNA levels of β-catenin and GSK-3β in the myocardial tissues. (**D**) The expression of β-catenin and GSK-3β in the myocardial tissues were measured using IHC staining (Scale bar = 500 *μ*m). (**E**) Influence of shZFAS1 on β-catenin and GSK-3β in the myocardial tissues. ^*^P < 0.05 compared with the group sham. ^#^*P* < 0.05 compared with the group MI.

Wnt/β-catenin pathway has been believed to be closely linked with the process of MI, and two key molecules, β-catenin and GSK-3β, were detected using western blotting, qRT-PCR and IHC staining methods. We found that significant decrease of β-catenin and increase of GSK-3β were observed in the group MI and MI+shNC (Figure 3A–3E) compared to group sham. However, the change trends of GSK-3β and β-catenin were remarkably reversed in the group MI+shZFAS1 (Figure 3A–3E).

### Inhibition of Wnt/β-catenin pathway remarkably reversed the influence of shZFAS1 on cardiac function

**Figure 4:**
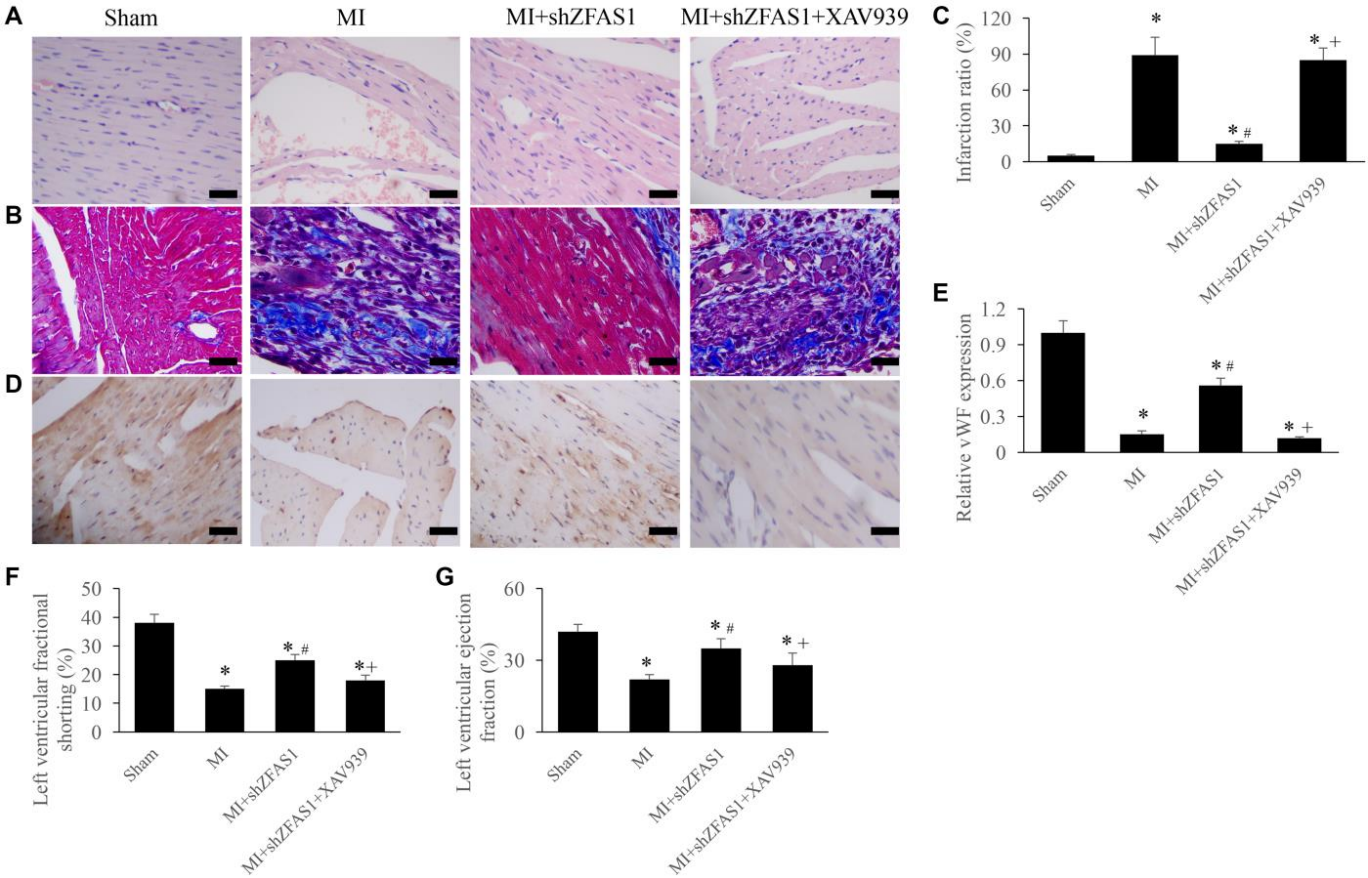
**Inhibition of Wnt/β-catenin pathway remarkably reversed the influence of shZFAS1 on cardiac function.** (**A**) Influence of XAV939 on the histological changes of MI rats myocardial tissues (Scale bar = 500 *μ*m). (**B**) Influence of XAV939 on the collagen deposition of MI rats myocardial tissues (Scale bar = 500 *μ*m). (**C**) Influence of XAV939 on the infarction ratio of MI rats myocardial tissues. (**D**) The expression of vWF in the MI rats myocardial tissues was measured using IHC staining (Scale bar = 500 *μ*m). (**E**) Influence of XAV939 on the vWF expression in the MI rats myocardial tissues. (**F**) Influence of XAV939 on the left ventricular fractional shortening of MI rats. (**G**) Influence of XAV939 on the left ventricular ejection fraction of MI rats. ^*^P < 0.05 compared with the group sham. ^#^*P* < 0.05 compared with the group MI.

To further unfold the potential influence of ZFAS1 on Wnt/β-catenin pathway, the inhibitor of Wnt/β-catenin pathway, XAV939, was used in the animal experiment. We found that MI+shZFAS1 could significantly reversed the changing trends of cardiac tissues morphological changes, infarction ratio, vWF expression, LVEF, and LVFS in the group MI (Figure 4A–4G). However, after simultaneous treatment with XAV939, significant increase of infarction ratio, and decrease of vWF, LVEF, and LVFS were observed compared with group MI+shZFAS1 (Figure 4A–4G). Therefore, ZFAS1 might regulate the process of MI through targeting Wnt/β-catenin pathway.

### Inhibition of Wnt/β-catenin pathway remarkably reversed the influence of shZFAS1 on CFs viability

**Figure 5:**
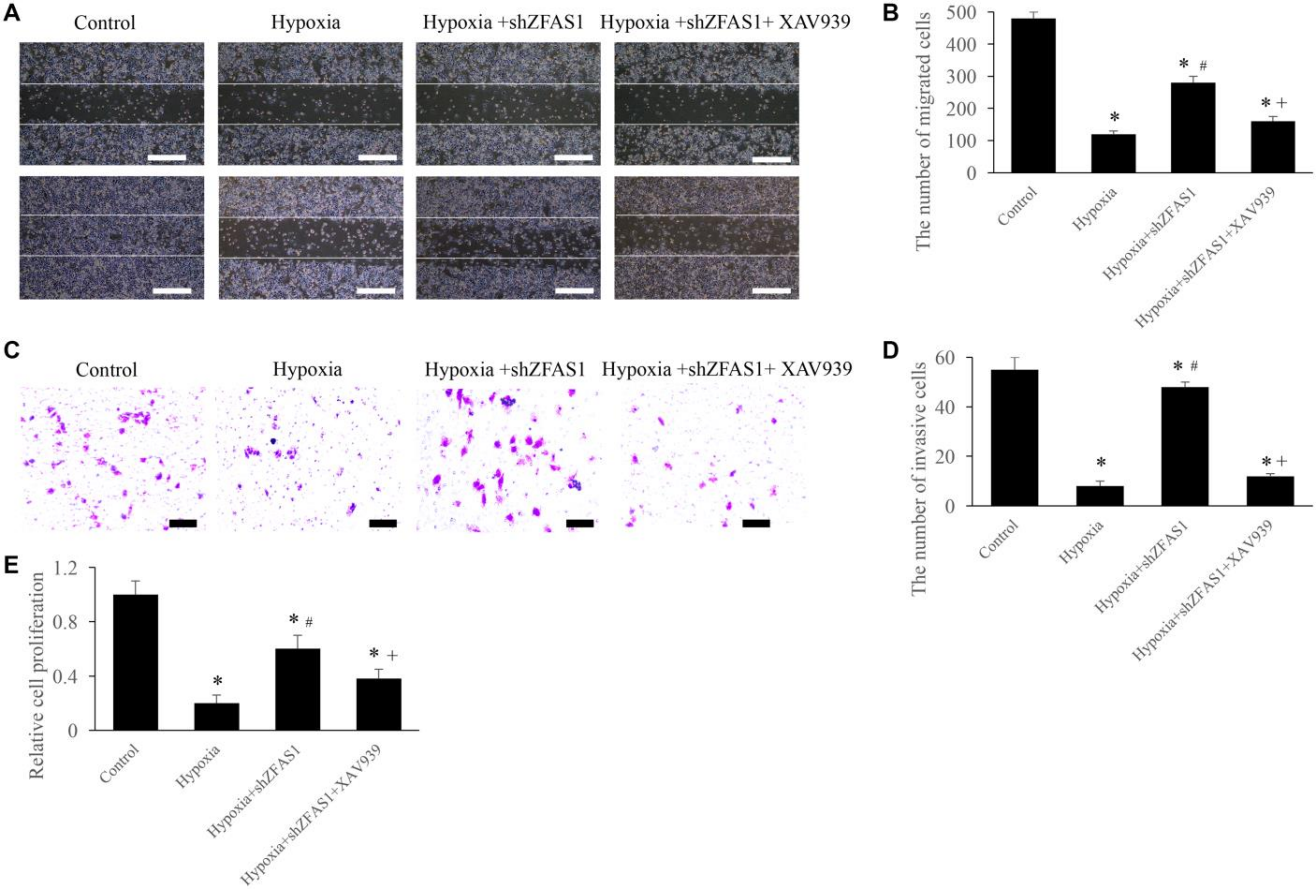
**Inhibition of Wnt/β-catenin pathway remarkably reversed the influence of shZFAS1 on CFs viability.** (**A**) Cell migration was measured using wound healing method (Scale bar = 500 *μ*m). (**B**) XAV939 markedly suppressed the ability of cell migration compared with group hypoxia+shZFAS1. (**C**) Cell invasion was measured using Transwell method (Scale bar = 200 *μ*m). (**D**) XAV939 markedly suppressed the ability of cell invasion compared with group hypoxia+shZFAS1. (**E**) XAV939 markedly suppressed the ability of cell proliferation compared with group hypoxia+shZFAS1. ^*^P < 0.05 compared with the group sham. ^#^*P* < 0.05 compared with the group hypoxia. ^+^*P* < 0.05 compared with the group hypoxia+shZFAS1.

The regulation of ZFAS1 on MI through Wnt/β-catenin pathway was further validated through *in vitro* experiments. We found that treatment with hypoxia+shZFAS1 significantly increased the migration, invasion, and proliferation ability of CFs compared to group hypoxia (Figure 5A–5E). However, treatment with hypoxia+shZFAS1+XAV939 remarkably inhibited the migration, invasion, and proliferation ability of CFs compared to group hypoxia+shZFAS1 (Figure 5A–5E). These findings further confirmed the conclusion that ZFAS1 might affect the process of MI via regulating Wnt/β-catenin pathway.

## DISCUSSION

MI is a serious cardiovascular disease that endangers human health and causes heavy social burden. The mortality and morbidity of MI still maintain high level despite great improvement of therapeutic methods [21]. In the late stage of MI, the proliferation and migration ability of myocardial fibroblasts increase and differentiate into myoid fibroblasts, which promotes MI and increases the degree of myocardial fibrosis [22].

Studies have found that LncRNA can participate in the regulation of myocardial cell apoptosis and myocardial fibrosis, which is closely linked with to occurrence and progression of MI. It was reported that microRNA-155 can regulate cardiac fibrosis through TGF-β1/Smad2 signaling pathway [23]. In MI model mice, the expression level of cyclin D1 was markedly suppressed and the level of p21 was promoted after inhibiting microRNA-155. LncRNA MALAT1 could regulate the activity of TGF- β1 through microRNA-145, and promote myocardial fibrosis and worsen cardiac function after MI. MALAT1 knockout can reduce cell proliferation, collagen production, and inhibit the activation of TGF- β1 induced by MI or angiotensin I, so as to reduce myocardial fibrosis and improve myocardial remodeling [24–26].

ZFAS1, as a member of LncRNA family, is expressed in both nucleus and cytoplasm, and it has about 70% conservation in human and mice [11]. ZFAS1 has been believed to regulate the progression of many kinds of tumors [27, 28]. Meanwhile, the roles of ZFAS1 in cardiovascular diseases were also reported. It is known that calcium overload caused by decreased SERCA2a could lead to myocardial dysfunction. Aa an endogenous inhibitor of SERCA2a, ZFAS1 could suppress the activity of SERCA2a, and further result in the impairment of cardiac contractile function [11].

Due to the remarkable low concentration in the serum of MI patients, ZFAS1 has been believed to be a maker for MI. However, the expression of ZFAS1 in the myocardium of MI mice was remarkably increased [11]. Our data indicated that ZFAS1 was significantly high expressed in the MI heart tissues and hypoxia cells. Further studies are required to unfold the role of ZFAS1 in MI process. LVFS and LVEF indicate left ventricular systolic function and ratio between output per stroke and volume of end diastolic ventricle, respectively. LVFS and LVEF are two sensitive parameters related with changes of myocardial function. In this study, knockdown of ZFAS1 significantly promoted LVFS, LVEF, and vWF expression, reduced infarction ratio, improved cardiac tissues remodeling (Figure 2).

Previous report indicated that knockdown of pigment epithelium-derived factor (PEDF) could remarkably suppress the levels of Wnt3a and β-catenin in myocardial tissue, but the down-regulation was significantly suppressed by the inhibitor of Wnt/β-catenin pathway. Silencing PEDF can suppress vascular endothelial injury by inhibiting Wnt/β-catenin signaling pathway, so as to reduce the cardiac function damage after MI [29, 30]. GSK-3β is a key downstream molecule of Wnt/β-catenin pathway. GSK-3β can promote ubiquitination and degradation of β-catenin through phosphorylation of Ser-33/37 site of β-catenin, and affect Wnt/β-catenin pathway mediated fibrosis. GSK-3β could promote the degradation of β-catenin and inhibit the production of β-catenin [31]. In this study, Wnt/β-catenin pathway was remarkably suppressed in the MI model, but knockdown of ZFAS1 reversed the trends compared with group MI. The Wnt/β-catenin pathway was activated by shZFAS1, the level of β- catenin was elevated, and GSK-3β was suppressed. Significant reduce of O_2_ supply is the main character of MI, which could lead to cell death, arrhythmia, and contractile dysfunction. Therefore, hypoxia cell model was also established in this study to confirm the conclusions of *in vivo* study.

In summary, we demonstrated that significant improvement of cardiac function in the MI rats was observed through knockdown of ZFAS1. In addition, we proved that ZFAS1 might regulate the process of MI through activating Wnt/β-catenin pathway. These findings were validated through MI rats and hypoxia cell models. This study uncovers the underlying regulation mechanism of ZFAS1 in MI process and provides a new insight for the prevention and treatment of MI.

## MATERIALS AND METHODS

### Cell culture and establishment of hypoxia cell model

Cardiac fibroblasts (CFs) were obtained from American Type Culture Collection (ATCC, USA). Normal CFs were incubated with DMEM containing 10% FBS in the incubator of 37°C and 5% CO_2_. Hypoxia cell model was established by incubating cells on the condition of 93% N_2_, 3% O_2_, and 4% CO_2_ for 24 h. Then, the cells were incubated with shZFAS1, shNC, and XAV939 for 12 h. Then, cells were used to measure cell proliferation, migration, and invasion. All experiments were approved by Ethic Committee of The Second Affiliated Hospital of Nanchang University (Approval reference number: 2020-016).

### Construction of ZFAS1 knockdown vector

AAV9 vector was used to carry a short RNA fragment to knockdown ZFAS1 (shZFAS1). The shZFAS1 and shNC were constructed by GenePharma Co., Ltd (Shanghai, China). Virus solution (5 × 10^13^ genome-containing particles) was used to treat rats through a caudal vein.

### Establishment of MI animal model

Wistar rats (male, 240–260 g, Charles River, China) were used in this study. The animals were kept in the condition of 25–28°C, 45–55% humidity with free access to food and water. The rats were divided randomly into different groups. MI model was established by performing left anterior descending ligation using 6.0 nylon. The rats were firstly anesthetized through intraperitoneal injection with xylazine (11 mg/kg) and ketamine (110 mg/kg). After shaving the chest of rats, a left parasternal incision was performed to open thoracic cavity. Then, heart was exposed, and left anterior descending was ligated for 1 h. Remarkable elevation of S-T segment in electrocardiograph indicated MI. Same operation was conducted for the sham group rats excluding the ligation of left anterior descending artery. 1 week after operation, rats were injected with shZFAS1, shNC, or XAV939. 2 weeks later, rats were sacrificed and heart tissues were collected for histological examination.

### Real-time polymerase chain reaction (RT-PCR)

RNA was isolated through TRIzol reagent (#15596026, Invitrogen, USA). 30 ng RNA was reverse-transcribed into cDNA with SuperScript™ II Reverse Transcriptase (#18064022, Invitrogen, USA). RT-PCR was performed using SYBR Premix Ex Taq™ II kit (Takara, China). The primers were listed as follows: (1) β-catenin: forward: 5′-TTCGCCTTCACTATGGA CTACC-3′ and reverse: 5′-GCACGAACAAGCAAC TGAACTA-3′; (2) GSK-3β: forward: 5′- CGAUUACACGUCUAGUAUA -3′ and reverse: 5′- UAUACUAGACGUGUAAUCG -3′; (3) GAPDH: forward: 5′-ACAACAGCCTCAAGATCATCAG-3′ and reverse: 5′-GGTCCACCACTGACACGTTG-3′. The mRNA level of the experimental group was determined by comparing the value of ΔΔCt.

### Western blot analysis

Tissues were lysed firstly, and protein content was detected using bicinchoninic acid kit (BCA, #P0012S, Beyotime, China) assay. Same amount of protein was separated by 10% SDS-PAGE and transferred to nitrocellulose membrane (Invitrogen, USA). The membrane was blocked with TBST solution (5% skim milk) for 2 h, and cultured with primary antibodies (1:800) at 4°C overnight. After washing twice using PBS, a secondary antibody (1:2000) was used to incubate proteins for 1 h. ImageJ software was applied to calculate the protein bands grey. The antibodies details were listed as follows: anti-beta Catenin antibody (ab32572, Abcam, Cambridge, UK), anti-GSK3 beta antibody (ab32391, Abcam, Cambridge, UK), and goat anti-Rabbit IgG (ab205718, Abcam, Cambridge, UK).

### Transwell assay

Cells (1 × 10^6^ each well) were seeded into the upper chamber, and 2 mL DMEM containing 15% FBS was added to the down chamber. The cells were cultured for 24 h, then 4% polyformaldehyde was used to fix cells for 20 min. After washing twice, Giemsa (Thermo Scientific, USA) was used to stain lower chamber. The invasive cells in the five fields were captured and analysed.

### Wound healing assay

The cells were diluted using DMEM and plated into a 6-well plate. When cell confluence grew to 70%, a 200 *μ*L pipette tip was applied to make a wound in the middle of plate. The cell number in the wound line was counted at 0 and 48 h.

### Ventricular function assessment

Transthoracic echocardiography was performed to evaluate the hemodynamic assessment of left ventricular function using a Xario ultrasound device (Toshiba, Japan). Rats were firstly anesthetized using xylazine (11 mg/kg) and ketamine (110 mg/kg). Then hemodynamic items were recorded through MP100-CE (BIOPAC Systems, USA).

### Histopathological analysis

The tissues were fixed with 4% paraformaldehyde for 24 h. Then, tissues were embedded with paraffin and cut into 8-μm thick slides. The tissues were stained through hematoxylin-eosin (HE) and Masson trichrome methods, respectively. Five fields were selected for analysis. An inverted optical microscope was used for analyzing.

### Immunohistochemistry staining

After de-paraffin, microwave heating was applied to repair antigen. Then, tissues were washed (twice, 5 min/time), and cultured with 5% H_2_O_2_ (5 min). After blocking with 10% goat serum, the slides were cultured with primary antibody at 4°C overnight. After washing (twice, 5 min/time), the sections were cultured with secondary antibody for 1 h. Then DAB regent was applied to incubate sections, and an inverted microscope was used for analyzing.

### Cell proliferation

MTT assay (#ST316, Beyotime, China) was applied to detect cell proliferation. Cells (1 × 10^5^) were plated into 96-well plate and cultivated for 24 h. After different treatments, cells were incubated with MTT reagent (20 μL) for 4 h, OD at 490 nm was detected.

### Statistical analysis

Data was presented by mean ± standard deviation (SD), and analyzed through SPSS 20.0. Student's *t*-test was applied to analyze results between two groups. Data more than 2 groups was calculated using one-way analysis of variance. *P* < 0.05 means statistically significant. All experiments were repeated at least 3 times.
